# The ‘Other’ in AWaRe

**DOI:** 10.1093/jacamr/dlae196

**Published:** 2024-12-03

**Authors:** Areeb Arshad, Clark D Russell, Barbara Moore, Simon Dewar

**Affiliations:** Medical Microbiology, Royal Infirmary of Edinburgh, 51 Little France, Old Dalkieth Road, Edinburgh EH16 4SA, UK; Medical Microbiology, Royal Infirmary of Edinburgh, 51 Little France, Old Dalkieth Road, Edinburgh EH16 4SA, UK; Centre for Inflammation Research, Institute for Regeneration and Repair, The University of Edinburgh, Edinburgh, UK; Medical Microbiology, Royal Infirmary of Edinburgh, 51 Little France, Old Dalkieth Road, Edinburgh EH16 4SA, UK; Medical Microbiology, Royal Infirmary of Edinburgh, 51 Little France, Old Dalkieth Road, Edinburgh EH16 4SA, UK; Clinical Infection Research Group, Western General Hospital, Edinburgh, UK

In 2017 (and revised in 2019, 2021 and 2023) the WHO committee on the selection and use of essential medicines developed the AWaRe (Access, Watch, Reserve) classification of antibiotics to provide a framework to monitor antibiotic use and support antimicrobial stewardship activities.^1^ In 2018 this WHO classification was adapted for national antibiotic stewardship polices by NHS England, and this adaptation is currently used throughout the UK (including Scotland).^2^ As well as the Access, Watch and Reserve categories there is an ‘Other’ category encompassing agents difficult to place in the standard groupings, for example antimycobacterials. In 2016 the ‘Other’ category accounted for 0.1% of antibiotics used in the acute hospital sector in NHS England.^2^

**Figure 1.**
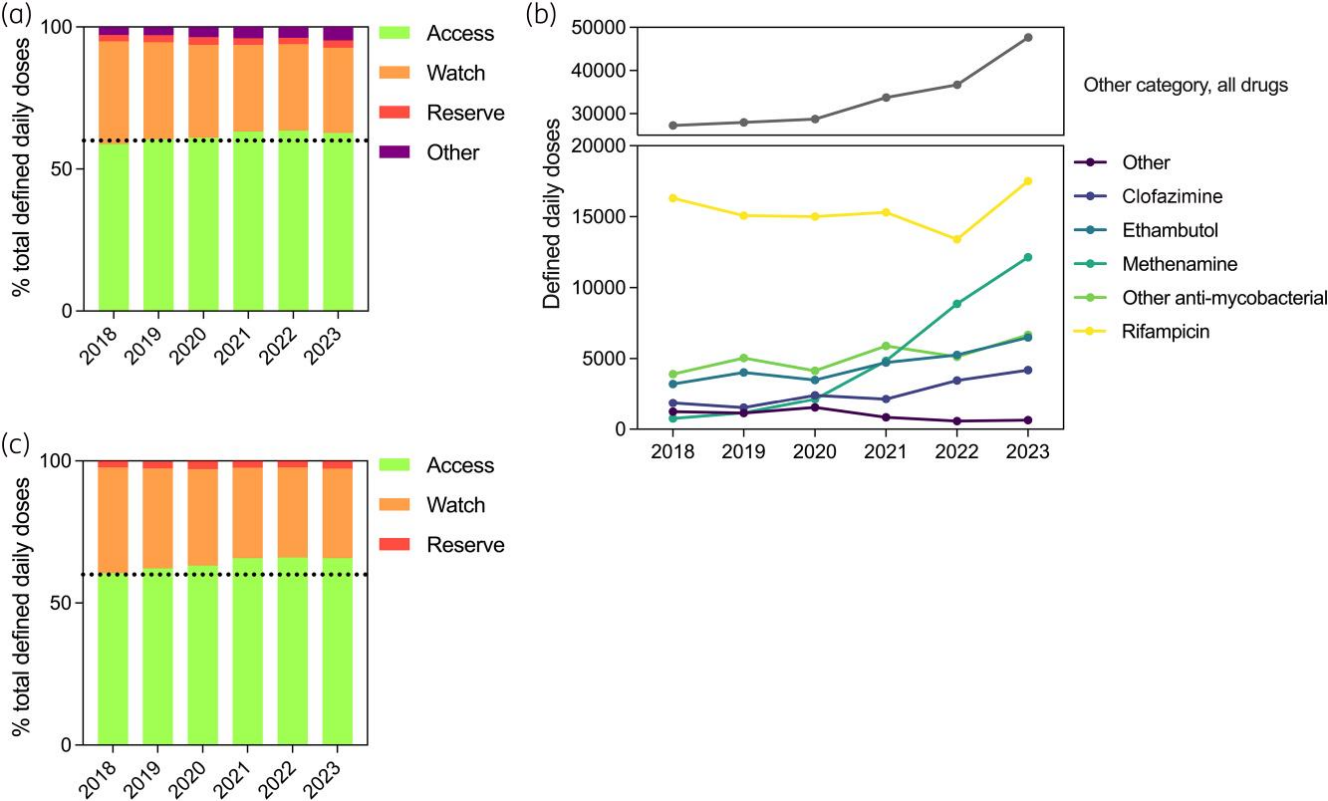
Antibiotics used in acute adult hospitals, NHS Lothian. (a) Percentage of antibiotic consumption in defined daily doses (DDDs) per category each year in NHS Lothian acute adult hospitals. The dotted line indicates 60% ‘Access’ use. (b) NHS Lothian acute adult hospitals consumption of all antimicrobials in the Other category over time (top). Trends in use in DDDs, of selected Other antimicrobials over time (bottom). Break in *y*-axis at 20 000 DDD. (c) The same graph as (a) with the ‘Other’ category excluded. Adult acute hospitals NHS Lothian include Royal Infirmary of Edinburgh, Western General Hospital and St John’s Hospital Livingstone. Access, Watch, Reserve and Other categories as per 2018 NHS England adaptation.^2^ All ‘Other’ agents used in NHS Lothian: clofazimine, cycloserine, demeclocycline, ethambutol, isoniazid, methenamine hippurate, pyrazinamide, rifampicin, Rifater, sulfadiazine and tinidazole.

In NHS Lothian Scotland, Access use in adult acute hospitals has steadily increased since 2018 (date of AWaRe implementation in the UK). Access use met the Scottish government national indicator that at least 60% of total antibiotic use in acute hospitals should be Access antibiotics by 2023 (total antibiotic use constituting Access, Watch, Reserve and Other) (Figure 1a). Looking further, Access use has plateaued in recent years, Watch use has declined, Reserve use is stable and Other use has increased. Within the Other category there has been an increase particularly in antimycobacterial agents and methenamine hippurate (Figure 1b). In Scotland, the incidence of infections caused by non-tuberculous mycobacteria (NTM) has increased,^3^ as has MDR-TB,^4^ meaning increased requirement for antimycobacterial agents. With regards to methenamine hippurate, a urinary antiseptic drug used in the prevention of recurrent urinary tract infections (UTIs), use of this in NHS Lothian has increased 16-fold since 2018 (Figure 1c). This coincides with the development of a local specialist urology service for recurrent UTI and methenamine hippurate featuring more prominently in guidance and practice, reflective of recent evolving evidence supporting its use as an alternative to antibiotics in preventing recurrent UTI.^5,6^

The recent UK National Action Plan target is to achieve 70% total use of antibiotics from the Access category across the human health system.^7^ An updated UK AWaRe categorization is yet to be published but will likely include updated Access, Watch, Reserve and Other categories. As well as monitoring for trends in Access, Watch and Reserve groups, it would be interesting to note trends in Other use. In 2023 ‘Other’ accounted for 4.7% of antibiotics used in adult acute hospitals NHS Lothian, whereas in 2016 it accounted for only 0.1% of antibiotics used in the acute sector in NHS England.^2^ It may be that NHS Lothian is an outlier with increased Other use; however, the increased use of agents such as methenamine hippurate is likely to be reflected UK-wide.

We also suggest that when analysing percentage Access use the denominator used should be closely examined, especially when the denominator may include non-antimicrobial agents such as methenamine hippurate. Locally we have found when comparing Access use with Access, Watch and Reserve use only (excluding the Other category), the proportion of Access use increases (e.g. in 2023, 65.8% Access versus Access, Watch and Reserve compared with 62.7% Access versus total use) (Figure 1c). This potentially illustrates continuing improvement in Access use as a proportion (by excluding the Other category in the denominator), and overall stewardship progression in line with national objectives, and could be represented accordingly. Stewardship goals should potentially focus on Access, Watch and Reserve antibiotics, with recognition that ‘Other’ categorization may be increasing and this may impact how Access use is monitored.
